# Nonpreserved amniotic membrane transplantation for bilateral toxic keratopathy caused by topical anesthetic abuse: a case report

**DOI:** 10.1186/1752-1947-4-262

**Published:** 2010-08-10

**Authors:** Ayse Asyali Altinok, Melike Balikoglu, Emine Sen, Kurtulus Serdar

**Affiliations:** 1 Eye Clinic I, Ulucanlar Eye Education and Research Hospital, Ankara, Turkey

## Abstract

**Introduction:**

Corneal damage associated with abuse of topical anesthetics is a rare clinic entity. Topical anesthetic abuse is one of the causes of ring keratitis. Ring keratitis is easily overlooked because it can mimic acanthamoeba keratitis or other infectious keratitis. The outcome is often poor, leading to persistent epithelial defects, corneal scarring, and perforations.

**Case presentation:**

We report the clinical presentation, diagnosis, and treatment of a 65-year-old Caucasian man, who worked as a health care worker, with bilateral toxic keratopathy caused by topical anesthetic abuse. Nonpreserved amniotic membrane transplantation was performed for both eyes of the patient.

**Conclusion:**

It is important to identify and treat patients who abuse topical anesthetics before permanent vision loss ensues. Nonpreserved amniotic membrane transplantation may be useful in relieving pain and improving corneal surface in anesthetic agent abusers.

## Introduction

Corneal damage associated with abuse of topical anesthetics has been reported by various sources [1-3]. Topical anesthetic abuse is one of the causes of ring keratitis [2]. This rare clinic entity is easily overlooked because it can mimic acanthamoeba keratitis or other infectious keratitis. The outcome is often poor, leading to persistent epithelial defects, corneal scarring, and perforations [1-3].

A case of bilateral toxic keratopathy caused by topical anesthetic abuse that was treated with nonpreserved amniotic membrane transplantation (NP-AMT) has been reported. To the best of our knowledge, this is the first report of NP-AMT use for the treatment of bilateral toxic keratopathy caused by topical anesthetic abuse.

## Case presentation

**Figure 1:**
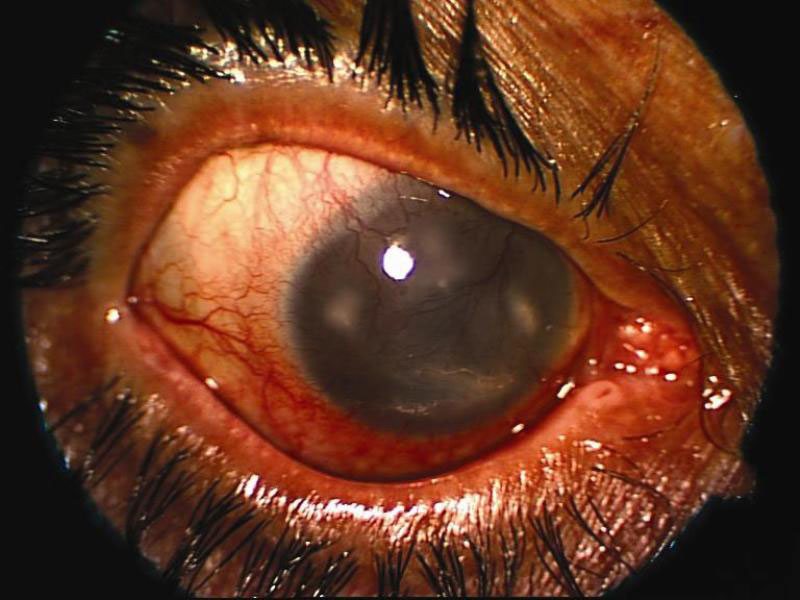
**Photograph demonstrating a central epithelial defect of 3 × 1 mm with mid-stromal ring infiltrates in the right eye**.

A 65-year-old Caucasian man, who worked as a health care worker, was admitted to our hospital with a history of severe eye pain, redness, and blurred vision in both eyes. His complaints had started with a mild eye itching six weeks prior to admission. He had a history of psychoactive substance carbamazepine and topical proparacaine abuse of three years. Our initial ocular examination showed bilateral intense conjunctival injection, corneal edema, diffuse corneal vascularization, and ring shaped stromal infiltration. There were also central epithelial defects of 3 × 1 mm with mid-stromal ring infiltrates in the right eye (Figure 1) and 6 × 8 mm with mid-stromal ring infiltrates in the left eye. Visual acuity of the right and left eyes was hand motions and finger counting at a distance of one meter, respectively.

**Figure 2:**
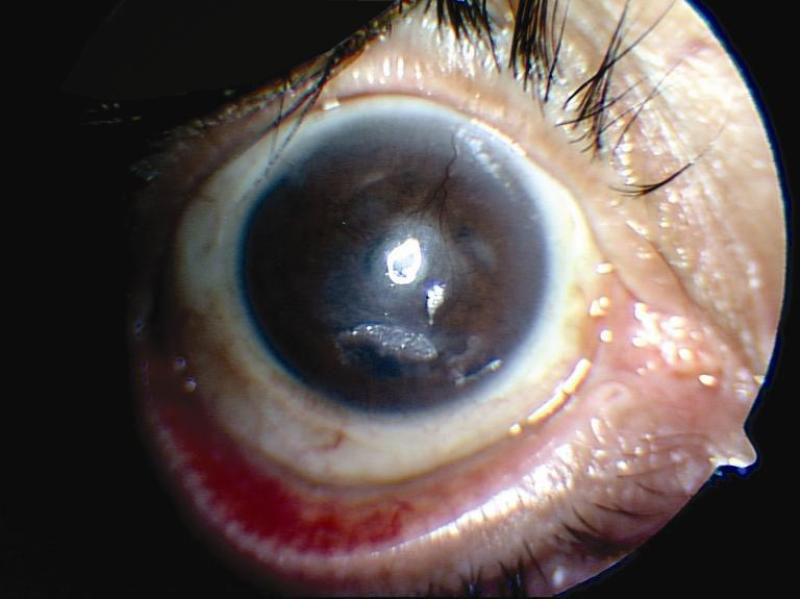
**After nonpreserved amniotic membrane transplantation, hypopyon was detected in the left eye**.

The cultures of corneal scrapings were negative. Proparacaine drops were discontinued, and our patient was prescribed preservative-free artificial tear drops and prophylactic topical antibiotic (ciprofloxacin 0.3%) five times a day. For his pain, oral indomethacin (75 mg three times a day) and topical ketorolac tromethamine (0.5% four drops a day) were added. Psychiatric counseling revealed psychoactive substance abuse and psychiatric disturbances. Despite medical treatment and conservative approach, the condition of our patient did not improve. Then, to achieve rapid epithelization, NP-AMT was planned for both eyes of our patient, as was previously described [4]. Initially, NP-AMT was used on the right eye. Owing to the pain in the right eye of our patient and persistent corneal epithelial defect decreased during the follow-up period, we performed NP-AMT on the left eye from another donor. At this stage, our patient's visual acuity was hand motions in both eyes. Three weeks after NP-AMT, a rapid regression of the external inflammatory signs, progressive clearing of the membrane, and a closed corneal epithelium were noted in the right eye. However, hypopyon was detected in the left eye (Figure 2). Repeat cultures of the corneal scrapings were negative. An ultrasound of this eye showed no vitreous infiltration. Because of suspected sterile hypopyon iritis, our patient was administered 100 mg hydrocortisone and 2.0 g ceftriaxon intravenously. Subsequently, the hypopyon resolved within three days. In the second week, systemic steroid use was tapered, and the use of antibiotic eye drops was ended. In the fifth week, our patient was caught trying to steal a bottle of proparacaine. The psychiatry clinic was consulted for further investigation and treatment. Because of poor compliance our patient was re-hospitalized and kept under close surveillance.

**Figure 3:**
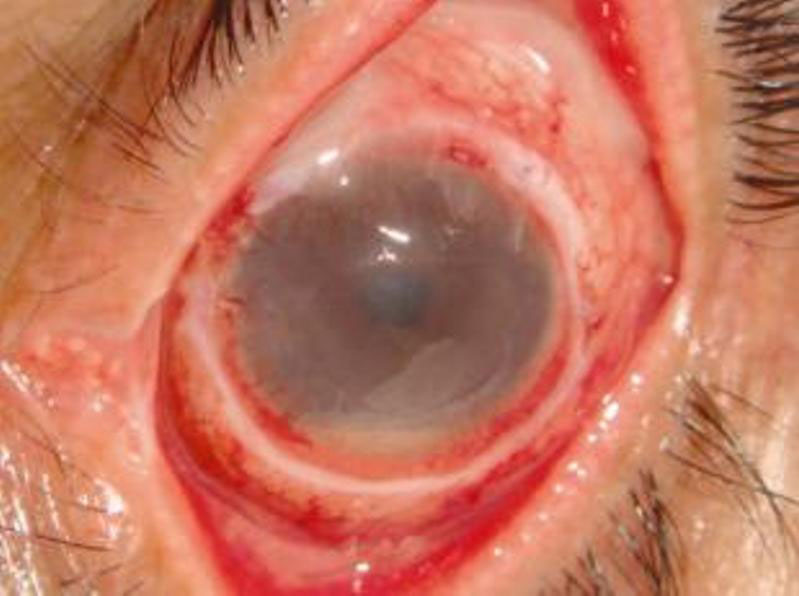
**In the final examination, the patient had no pain and no epithelial defect in the right eye**.

**Figure 4:**
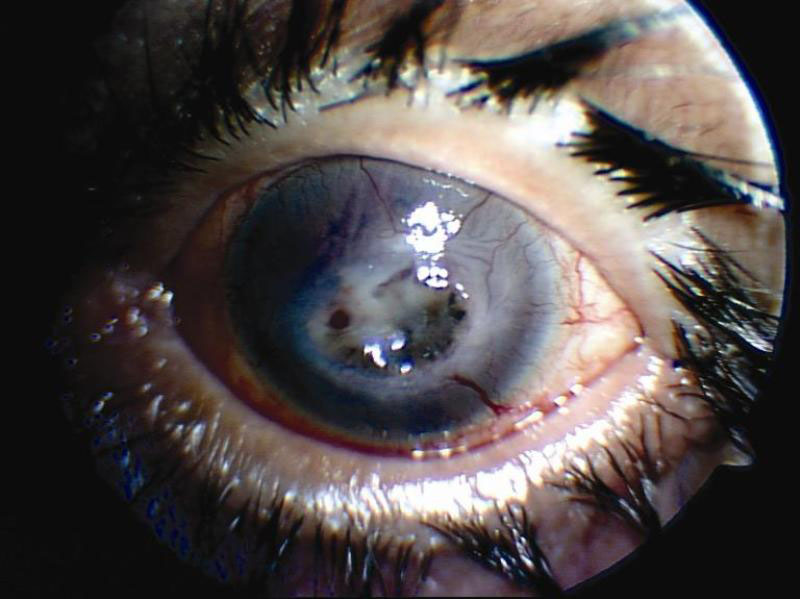
**In the final examination, he had impending corneal perforation in the left eye**.

At two months, our patient had no pain and no epithelial defects in the right eye (Figure 3). He had impending corneal perforation in the left eye. The visual acuity in the right eye was finger counting at a distance of four meters with residual corneal scarring, and in the left eye, it was limited to finger counting at a distance of one meter. Our patient was referred to the eye bank for penetrating keratoplasty, which was required to treat corneal perforation in his left eye (Figure 4).

## Discussion

Topical anesthetic abuse is a serious disorder, which involves persistent epithelial defects, corneal stromal ring infiltrates, anterior segment inflammation, disproportionate pain, visual loss, and a history of psychoactive substance abuse [1-3]. Rosenwasser *et al. *determined poor visual acuity in six patients [3]. Another study demonstrated that all topical anesthetics caused a reduction in the rate of epithelial healing in an animal model [4]. Management of this disorder depends on the discontinuation of the anesthetic agent, which is very difficult for the patients because of psychoactive substance abuse. Topical anesthetic abuse occurs mostly in patients with access to medication, for example nurses and pharmacists [2,3]. Similarly, our patient was a health care worker. Since patient compliance to conservative approaches was poor, we chose to perform NP-AMT in our patient.

This surgery has been successfully performed many times at our institution, and no post-operative intra-ocular inflammation has been encountered to date [5]. Hypopyon occurred rarely after amniotic membrane transplantation for ocular surface disorders [6]. Most types of anterior uveitis are sterile inflammatory reactions [7]. A hypopyon was determined in our patient's left eye. An ultrasound of this eye showed no vitreous infiltration. In light of these findings, our patient's condition was considered to be a case of sterile inflammation, and additional intravenous steroid use was preferred. Then, the hypopyon resolved within three days.

Clinical evidence indicates that amniotic membrane cells do not express histocompatibility (HLA) antigens A, B, C, or DR. Although the amniotic membrane preparation, the surgical procedure applied, and the surgeon were the same, hypopyon occurred in one eye after NP-AMT. Contrary to what has been suggested in the literature [6], we used amniotic membrane from different donors. This may account for local immunoreaction after NP-AMT.

The outcome of topical anesthetic abuse cases is poor because of continued drug use after keratitis commences [1,3]. This is because the attending physician may not suspect drug abuse and/or dishonesty on the part of the patient. Anesthetic abusers frequently continue to self-administer anesthetic agents, often covertly, even when informed of the consequences of their actions. Thus, long-term anatomical and functional results are very poor. Despite NP-AMT, in our patient, the right eye healed with residual corneal scarring, and the left eye required a penetrating keratoplasty.

Psychiatric consultation is extremely helpful and should be considered in the management of these patients. We recommend that the patients be hospitalized and treated under close supervision. It is important to identify and treat patients who abuse topical anesthetics before permanent vision loss develops.

## Conclusions

It is important to identify and treat patients who abuse topical anesthetics before permanent vision loss ensues. In addition, close medical supervision and psychiatric consultation should be considered. As a final option, NP-AMT may be considered in relieving pain and improving corneal surface in resistant anesthetic agent abusers. However, the efficiency of NP-AMT cannot be determined based on this single case alone. Further studies, which will investigate the changes after NP-AMT, compare its clinical outcomes, and evaluate safety and efficacy of NP-AMT to treat anesthetic abuse keratopathy, are needed.

## Abbreviations

NP-AMT: nonpreserved amniotic membrane transplantation.

## Consent

Written informed consent was obtained from the patient for publication of this case report and any accompanying images. A copy of the written consent is available for review by the Editor-in-Chief of this journal.

## Competing interests

The authors declare that they have no competing interests.

## Authors' contributions

AAA and MB were major contributors in writing the manuscript and reviewed the patient's notes. EMS drafted and revised the manuscript critically for important intellectual content. KS collected the psychiatric data, observed the patient closely. All the authors read and approved the final manuscript.
